# SNAP-25b-deficiency increases insulin secretion and changes spatiotemporal profile of Ca^2+^oscillations in β cell networks

**DOI:** 10.1038/s41598-017-08082-y

**Published:** 2017-08-10

**Authors:** Teresa Daraio, Lidija Križančić Bombek, Marko Gosak, Ismael Valladolid-Acebes, Maša Skelin Klemen, Essam Refai, Per-Olof Berggren, Kerstin Brismar, Marjan Slak Rupnik, Christina Bark

**Affiliations:** 1 The Rolf Luft Research Center for Diabetes and Endocrinology, Department of Molecular Medicine and Surgery, Karolinska Institutet, 171 76 Stockholm, Sweden; 2 Institute of Physiology, Faculty of Medicine, University of Maribor, SI-2000 Maribor, Slovenia; 3 Department of Physics, Faculty of Natural Sciences and Mathematics, University of Maribor, SI-2000 Maribor, Slovenia; 4 Center for Physiology and Pharmacology, Medical University of Vienna, A-1090 Vienna, Austria

## Abstract

SNAP-25 is a protein of the core SNARE complex mediating stimulus-dependent release of insulin from pancreatic β cells. The protein exists as two alternatively spliced isoforms, SNAP-25a and SNAP-25b, differing in 9 out of 206 amino acids, yet their specific roles in pancreatic β cells remain unclear. We explored the effect of SNAP-25b-deficiency on glucose-stimulated insulin release in islets and found increased secretion both *in vivo* and *in vitro*. However, slow photo-release of caged Ca^2+^ in β cells within pancreatic slices showed no significant differences in Ca^2+^-sensitivity, amplitude or rate of exocytosis between SNAP-25b-deficient and wild-type littermates. Therefore, we next investigated if Ca^2+^ handling was affected in glucose-stimulated β cells using intracellular Ca^2+^-imaging and found premature activation and delayed termination of [Ca^2+^]_*i*_ elevations. These findings were accompanied by less synchronized Ca^2+^-oscillations and hence more segregated functional β cell networks in SNAP-25b-deficient mice. Islet gross morphology and architecture were maintained in mutant mice, although sex specific compensatory changes were observed. Thus, our study proposes that SNAP-25b in pancreatic β cells, except for participating in the core SNARE complex, is necessary for accurate regulation of Ca^2+^-dynamics.

## Introduction

A controlled insulin secretion from β cells in the islets of Langerhans is essential to preserve healthy levels of blood glucose during basal and stimulated conditions^1^. Glucose-driven insulin secretion is mediated by SNARE proteins (*S*oluble *N*SF-attachment *P*rotein (SNAP) *RE*ceptors; NSF, N-ethylmaleimide-sensitive fusion protein) and requires an increase in cytosolic Ca^2+^ ^2, 3^. When β cells sense, import and metabolize glucose from the blood, molecular processes increase cytosolic levels of Ca^2+^, which activate the SNARE machinery mediating insulin granule fusion with the plasma membrane. Glucose stimulation of β cells normally induces a biphasic response of insulin secretion, consisting of an initial fast first phase lasting a few minutes, followed by a sustained long-lasting second phase characterized by pulsatile insulin release^1, 4, 5^.

The excitable β cell expresses essentially the same repertoire of SNARE proteins as neuroendocrine and neuronal cells^2, 3, 6^. The SNARE complex includes the plasma membrane proteins syntaxin and synaptosomal-associated protein of 25 kD (SNAP-25), and the vesicular protein VAMP/synaptobrevin. SNAP-25 is essential for stimulus-dependent exocytosis and exists as two isoforms, SNAP-25a and SNAP-25b. Alternative splicing between two exons 5 in the *Snap25* gene results in two proteins, differing in only 9 out of 206 amino acids^7–9^. Messenger RNAs for both SNAP-25 isoforms are expressed in primary pancreatic β cells and both variants support insulin secretion^10, 11^. The functional difference between the two isoforms is not fully understood, however, in mouse brain SNAP-25b forms more stable SNARE complexes than SNAP-25a^12^. Furthermore, in embryonic SNAP-25-deficient chromaffin cells, introduction of exogenous SNAP-25b induces a larger pool of primed vesicles than SNAP-25a, resulting in a higher burst of catecholamine secretion after stimulation^13^. Additionally, SNAP-25 together with syntaxin binds to the synprint site of voltage-dependent calcium channels, VDCC, potassium channels and G-protein-coupled receptors^14–22^. Thus, SNAP-25 plays also a role in the regulation of Ca^2+^ dynamics and membrane potential in β cells.

Controlled alterations of intracellular Ca^2+^ concentrations, [Ca^2+^]_*i*_, play a central role in insulin secretion from β cells. The first phase of insulin release is dependent on local Ca^2+^-influxes through VDCCs which recruit Ca^2+^ from endoplasmic reticulum. The sustained long-lasting second phase of rhythmic insulin release is also dependent on a more complex simultaneous intracellular signaling, including changes in ATP levels, phosphoinositides and the release of Ca^2+^ from intracellular stores^23, 24^. In healthy islets there exists also an extensive electrical coupling between individual β cells mediated through gap junctions, and these intercellular contacts are connecting hundreds of β cells into a functional network^25–28^. As a result, the dynamics of electrical activity, [Ca^2+^]_*i*_ oscillations and pulsatile insulin secretion are collectively controlled throughout the islet^29^. Alterations in islet morphology as well as in connexin36-dependent intercellular communication via gap junctions can result in loss of [Ca^2+^]_*i*_ coordination, which leads to an impairment of the normal pulsatile pattern of insulin secretion^4, 26, 30–32^. In several mouse models of diabetes^26, 32, 33^, in connexin36-null mouse models^30, 31^ and also in humans with prediabetes^34^ it has been shown that loss of synchronization in [Ca^2+^]_*i*_ oscillations is accompanied by a disruption of glucose sensitivity and impairment of the normal oscillatory pattern of insulin secretion.

Recently, we demonstrated that a genetically engineered mouse mutant expressing normal levels of SNAP-25 but without expressing the SNAP-25b isoform, developed metabolic impairments such as obesity, hyperglycemia, dyslipidemia, adipocyte hypertrophy and liver steatosis, a phenotype resembling the metabolic syndrome which was dramatically exaggerated when combined with a high fat/high carbohydrate diet intervention^35^. Here we have investigated how the absence of SNAP-25b influenced insulin secretion, as impaired insulin exocytosis by itself can act as a triggering factor for developing disease. We have analyzed the effect of SNAP-25b-deficiency during acute glucose-stimulated insulin secretion, gross islet morphology, Ca^2+^-dependent exocytosis in individual β cells and glucose-dependent β cell network activity.

## Results

### Islets from SNAP-25b-deficient mice secrete more insulin

**Figure 1:**
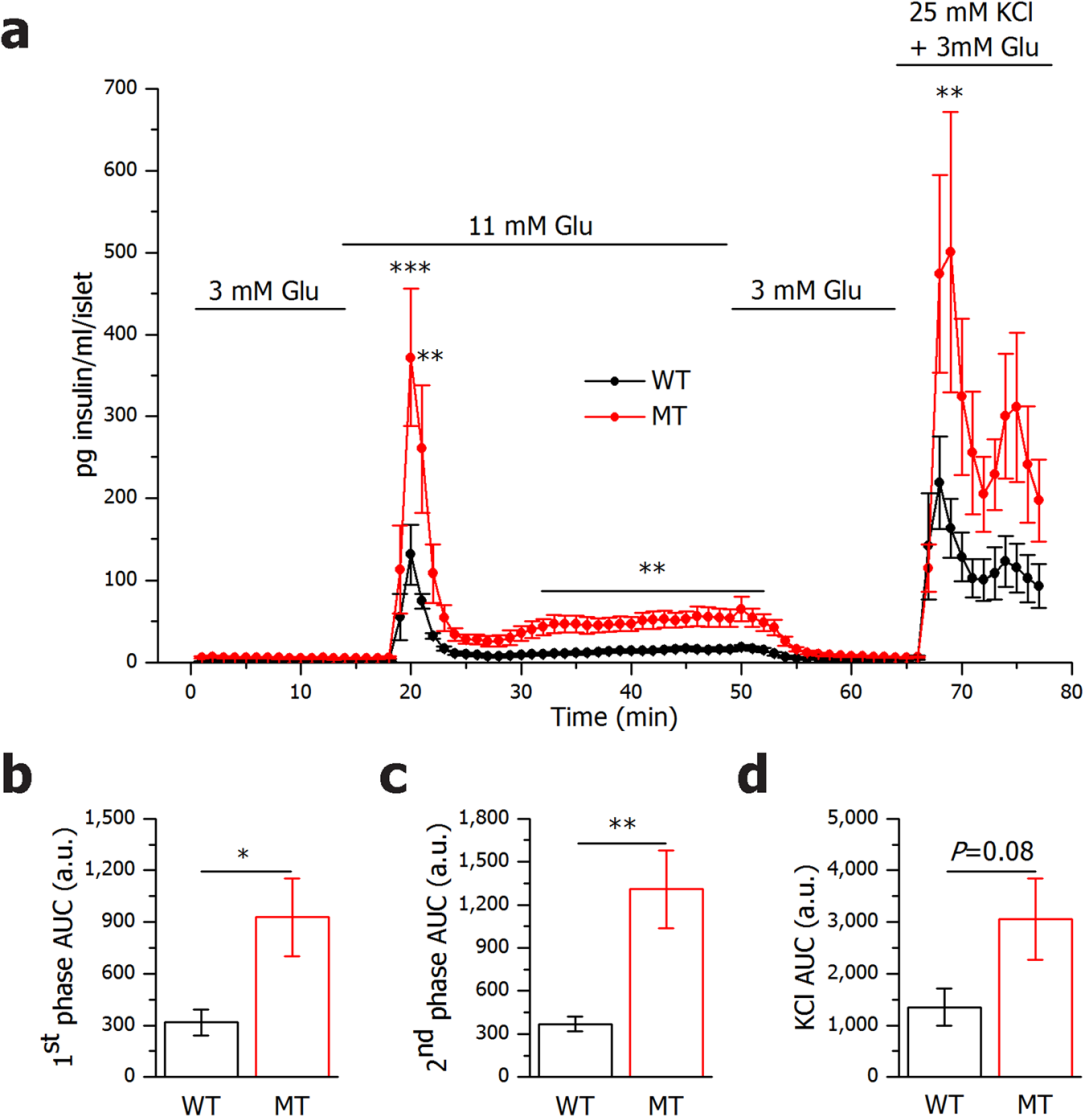
SNAP-25b-deficient islets secrete more insulin than wild-type littermates in response to glucose. Glucose-induced insulin release was measured in isolated pancreatic islets from male and female 12 week old wild-type (WT) and SNAP-25b-deficient (MT) mice (**a**). AUC was calculated for the first (**b**) and second (**c**) phase of insulin secretion as well as for KCl stimulation (**d**). *n* = 7–8 animals per group. WT mice, black circles and bars; MT mice red circles and bars. Data are represented as mean ± SEM, *P < 0.05, **P < 0.01, ***P < 0.001.

We first investigated the role of SNAP-25b-deficiency during glucose-stimulated insulin secretion in isolated pancreatic islets (Fig. 1). As shown in Fig. 1a, glucose stimulation resulted in an overall increased insulin secretion in SNAP-25b-deficient (MT) mice compared to their wild-type (WT) littermates. The area under the curve (AUC) was calculated for the first (Fig. 1b) and second phase (Fig. 1c) of insulin secretion and in MT mice the AUC during both phases was significantly increased compared to WT mice. KCl depolarization had a greater effect on insulin secretion in MT compared to WT mice although it was not significantly different (*P* = 0.08) (Fig. 1d). A similar trend was observed also when female and male mice were analyzed separately (Supplementary Fig. 1).

**Figure 2:**
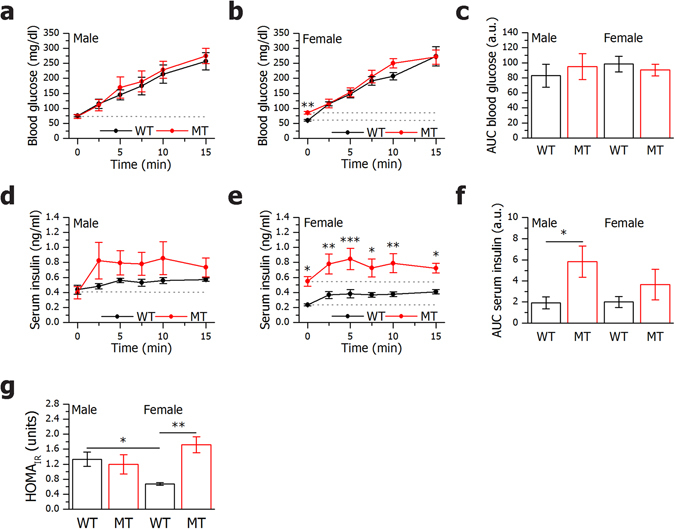
*In vivo* glucose tolerance tests demonstrate increased insulin secretion in SNAP-25b-deficient mice. After 12 h starvation, male and female 11 week old WT and MT mice received an *i.p*. glucose injection (2 g/kg). Blood glucose and serum insulin in males (**a**,**d**) and females (**b**,**e**) were measured during 15 min after glucose injection. AUC for blood glucose (**c**) and serum insulin (**f**) were calculated using basal levels as baseline (baselines used for calculations are indicated with dotted lines). HOMA_IR_ was calculated for all experimental groups (**g**). *n* = 5–6 animals per group. WT mice, black circles and bars; MT mice red circles and bars. Data are represented as mean ± SEM, *P < 0.05, **P < 0.01, ***P < 0.001.

To further confirm whether the facilitated insulin release found *in vitro* was also present *in vivo*, we focused on the secretory response within the first 15 min of a glucose tolerance test (GTT) (Fig. 2). Serum levels of insulin in MT mice challenged with glucose were higher compared to WTs at 2.5, 5, 7.5, 10 and 15 min after glucose injection (Fig. 2d–e). MT mice had a tendency to respond to glucose with a faster increase in serum insulin (Fig. 2d–e) and AUC above the resting serum insulin level was higher for MTs (Fig. 2f). Blood glucose levels and AUC above the basal glucose levels were comparable in all groups tested (Fig. 2a–c). WT females had consistently lower serum insulin levels compared to WT males and therefore MT females were hyperglycemic and hyperinsulinemic already at basal conditions compared to WT females (Fig. 2b,e). In fact, the WT females were found to be more sensitive to insulin compared both to WT males and MT females when the HOMA_IR_ was calculated (Fig. 2g). Furthermore, preliminary results from insulin tolerance tests support the HOMA_IR_ measurements (data not shown).

Taken together, SNAP-25b-deficiency increased glucose-stimulated insulin secretion both *in vitro* and *in vivo*.

### β cell hyperplasia in islets from SNAP-25b-deficient mice

**Figure 3:**
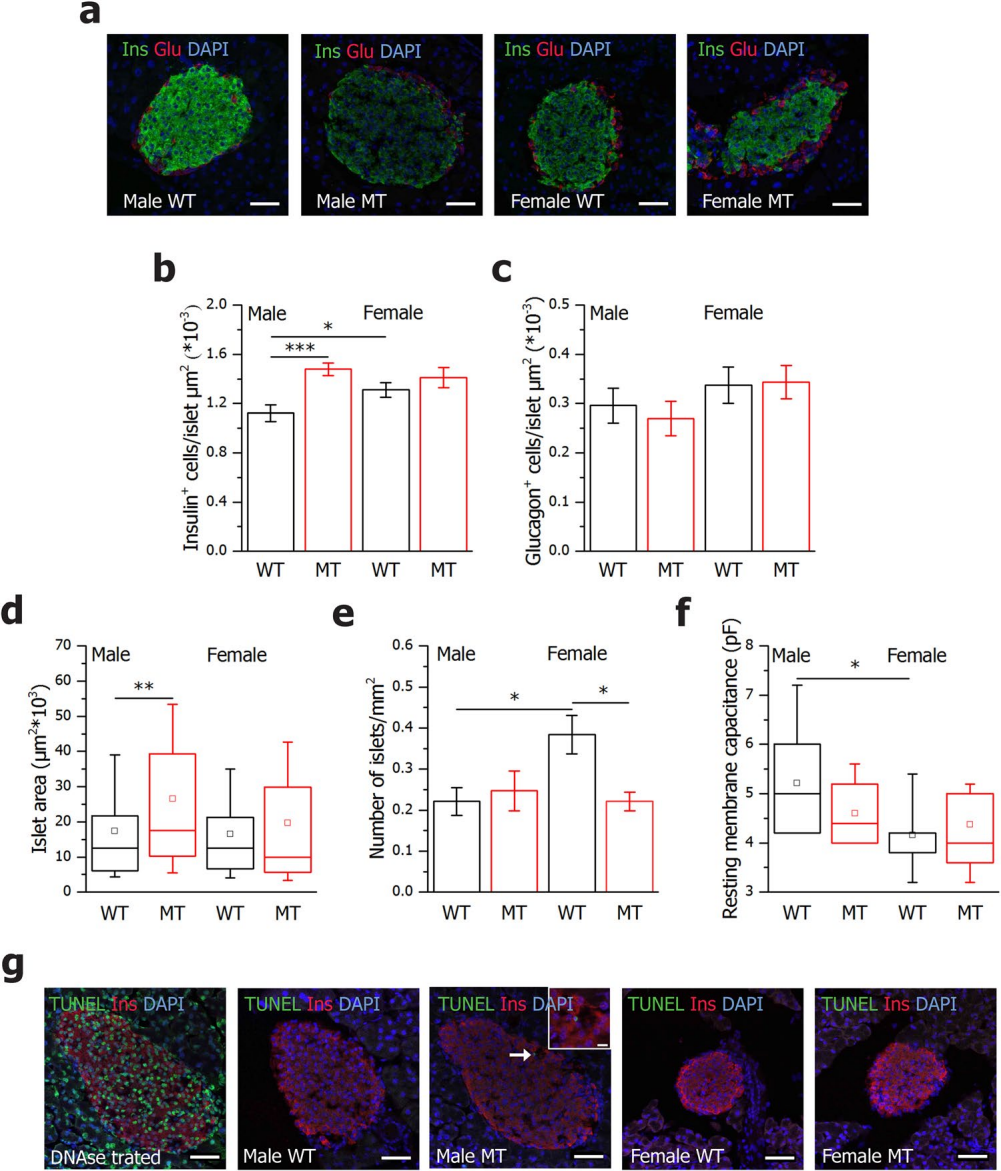
Male SNAP-25b-deficient islets are larger than WT littermates and have an increased number of β cells. Pancreatic sections from male and female 12 week old WT and MT mice were immunolabeled for insulin (green) and glucagon (red). Nuclei were detected with DAPI (blue) (**a**). Number of insulin-positive cells (**b**) and glucagon-positive cells (**c**) per islet was calculated in 3 islets from each mouse (*n* = 4 for each group). The islet areas are represented as box plots for male and female mice (**d**). The number of islets per pancreatic section area was calculated in male and female mice (**e**). Resting membrane capacitance of β cells (**f**), WT males, *n* = 2, 10 cells, MT males, *n* = 2, 11 cells, WT females, *n* = 4, 17 cells, MT females, *n* = 2, 11 cells. TUNEL staining of pancreatic sections from male and female 12 week old WT and MT mice (**g**). Nuclei were stained with DAPI (blue), β cells with anti-insulin antibody (red), and apopotic cells in green. A DNAse I treated section was added in the experiment as positive control (*n* = 3 for each experimental group). Only one β cell was found positive for TUNEL in a MT male islet (inset). The box plots (**d**,**f**) indicate the interval within 25th and 75th percentiles, lines within the boxes indicate the medians, and the small squares stand for the mean values. The whiskers denote the interval within 10th and 90th percentiles. Bar charts are represented as mean ± SEM (**b,c,e**). WT mice, black bars; MT mice red bars. Scale bar 50 µm (inset 10 µm). *P < 0.05, **P < 0.01, ***P < 0.001.

To address the issue of the aforementioned higher insulin secretion after glucose challenge in MT animals, we first explored the appearance of insulin- and glucagon-expressing cells in pancreatic sections by fluorescence immunohistochemistry (Fig. 3a). In islets of SNAP-25b-deficient mice a characteristic spatial distribution of α and β cells as in their WT littermates was found (Fig. 3a). Further analyses showed that MT males had an increased number of insulin-positive cells compared to WTs (Fig. 3b) and the islet size in MT males was significantly larger compared to WT males (Fig. 3d). WT females showed increased number of insulin-positive cells compared to WT males (Fig. 3b), although the individual β cells in the islets of WT females were smaller in size, as determined by membrane capacitance measurements (Fig. 3f). No significant differences were detected in the number of α cells per islet (Fig. 3c). Furthermore, the total number of islets per square millimeter in pancreatic sections was counted and MT females had fewer islets compared to WT females, whereas no difference was present in males (Fig. 3e). WT females were found to have a significantly increased number of islets compared to WT males (Fig. 3e). Although, no signs of an increased rate of apoptosis were found in β cells in MT mice compared to WT mice (Fig. 3g).

In conclusion, MT males displayed β cell hyperplasia which partly can explain the increased insulin release while MT females did not show any major morphological changes of the islets.

### SNAP-25b-deficiency does not affect Ca^2+^-sensitivity or rate of exocytosis

**Figure 4:**
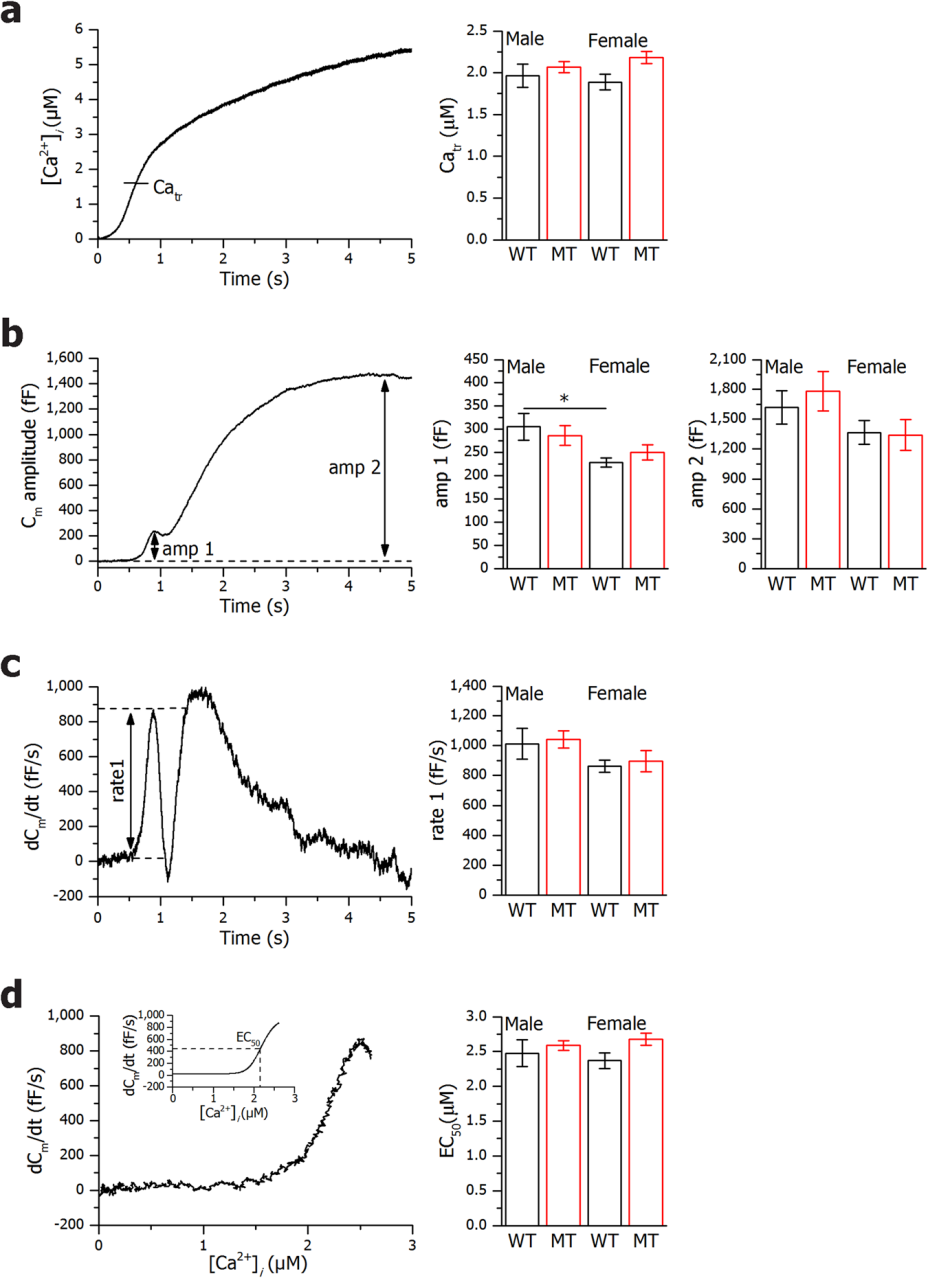
Slow photo-release of caged Ca^2+^. Slow photo-release of Ca^2+^ produced a ramp-like increase in [Ca^2+^]_*i*_ as shown in a representative β cell (left panel). Panel on the right displays calcium concentration measurements at which membrane capacitance was triggered for all experimental groups (**a**). After reaching the threshold value of [Ca^2+^]_*i*_ (Ca_*tr*_) a biphasic increase in membrane capacitance (*C*_*m*_) is observed, with the first phase reaching maximal amplitude within the first second after the initiation (amp1) and the second phase reaching maximal amplitude at amp2 (**b**). On the right panel amp1 and amp2 measurements for all experimental groups are shown (**b**). Time derivatives of *C*_*m*_ amplitudes are presented in left panel (**c**), with maximal rate of the first phase (rate1). In the right panel, rate1 measurements for all experimental groups are shown (**c**). The rate of the *C*_*m*_ change shown in panel d shows saturation kinetics when plotted versus [Ca^2+^]_*i*_ with high cooperativity and half-effective [Ca^2+^]_*i*_ (EC_50_) (**d**). The inset shows a Hill function fit through the Ca^2+^-dependence data (**d**). In the right panel, EC_50_ measurements for all experimental groups are shown (**d**). WT males, *n* = 2, 10 cells, MT males, *n* = 2, 11 cells, WT females, *n* = 4, 17 cells, MT females, *n* = 2, 11 cells. Bar charts are represented as mean ± SEM. WT mice, black bars; MT mice red bars. *P < 0.05.

To explore if the increased insulin secretion found in SNAP-25b-deficient β cells was dependent on less stable SNARE complexes that could facilitate exocytosis we performed experiments with slow photo-release of caged Ca^2+^ (Fig. 4). Unexpectedly, there were no significant differences either in Ca^2+^-sensitivity, amplitude or rate of exocytosis between WT and MT β cells when [Ca^2+^]_*i*_ was elevated (Fig. 4a–d). The Ca^2+^ concentration triggering exocytosis (Ca_*tr*_) was found to be around 2 μM for all experimental groups (Fig. 4a) coupled to a similar raise in membrane capacitance (Fig. 4b). Noteworthy is that amp1 (the maximum amplitude within the first second of exocytosis) was significantly lower in WT females compared to WT males, likely reflecting the smaller β cell size. Finally, no difference was found in the kinetic parameters rate1 and EC_50_ (Fig. 4c–d).

Thus, even if SNAP-25b forms more stable SNARE complexes than SNAP-25a^12^, we could conclude that SNAP-25b-deficiency did not change the Ca^2+^-sensitivity or efficacy of SNARE-mediated Ca^2+^-dependent exocytosis.

### SNAP-25b-deficiency increases premature Ca^2+^-activity in β cell subgroups after glucose stimulation

SNARE proteins, in particular SNAP-25 and syntaxin have previously been shown to interact both with VDCC via the synprint site and with different subtypes of potassium channels^16–22^. As the increased insulin secretion in SNAP-25b-deficient mice apparently was not due to changed stability of the exocytotic SNARE complex *per se*, we therefore instead explored if the increased insulin secretion could depend on altered regulation of Ca^2+^ dynamics. We monitored Ca^2+^-oscillations in β cells from acute pancreatic slices with confocal functional multicellular Ca^2+^ dynamics imaging. Stimulation with 12 mM glucose initiated the activation phase with its characteristic progressive recruitment of active β cells (Supplementary Fig. 2a–d)^27^. The recruitment process was found to be rather heterogeneous with delays to first responses spanning from 1–6 min, as shown in Supplementary Fig. 2e–f. It appears that in MT islets, small subgroups of cells became active earlier than in WT islets. This observation was quantified by showing the fraction of cells that were activated before the 70% of the mean activation value in a given islet (Supplementary Fig. 2g–h).

Seemingly, in SNAP-25b ablated islets, the fraction of β cells responding prematurely to glucose simulation was increased compared to WT islets, reflected by less regulated and faster Ca^2+^ responses.

### SNAP-25b-deficiency results in delayed β cell deactivation after glucose removal

The lack of coordination between β cells in MT islets likely affects the pattern of β cell deactivation after removal of stimulatory glucose concentrations. The activity of β cells progressively faded out and the Ca^2+^ concentration returned from the sustained plateau with superimposed oscillations back to the pre-stimulatory level as featured in Supplementary Fig. 3a–d. First cells became inactive around 2–3 min after decrease in glucose concentration, and the duration of the deactivation interval differed substantially from islet to islet, and could be even longer than 20 min. In females the variability was more pronounced than in males (Supplementary Fig. 3e–f). To further characterize the deactivation process, we calculated the deactivation times (Supplementary Fig. 3g–h), which revealed that in males the deactivation process of MT islets was significantly longer than in WTs.

In brief, in SNAP-25b-deficient β cells there was a delay in the deactivation process leaving the cells with increased [Ca^2+^]_*i*_ for a prolonged time after removal of stimuli compared to WT littermates.

### SNAP-25b-deficiency results in less synchronized Ca^2+^-oscillations

**Figure 5:**
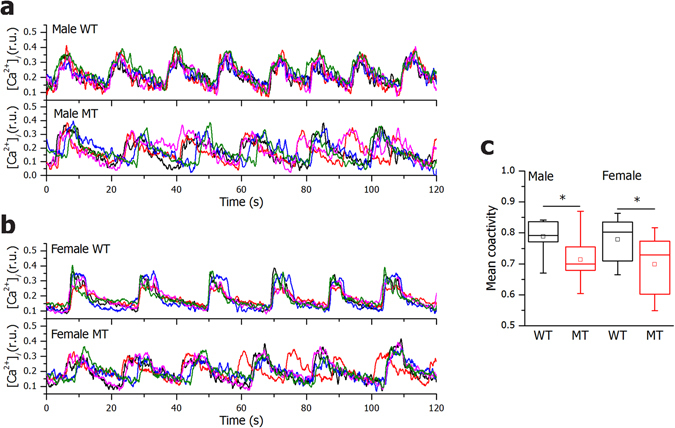
SNAP-25b-deficient β cells display less synchronized Ca^2+^ oscillations. Five typical [Ca^2+^]_*i*_ traces recorded during 12 mM glucose stimulation in a given slice from 12 week old WT and MT males (**a**) and WT and MT females (**b**). Mean coactivity coefficient pooled from all slices in a given sub-group for males and females (**c**). WT males, *n* = 4, 8 islets, MT males, *n* = 4, 9 islets, WT females, *n* = 3, 11 islets, MT females, *n* = 5, 11 islets. The box charts are defined in the same way as in Fig. 3. WT mice, black bars; MT mice red bars. *P < 0.05.

After 7–15 min of exposure to 12 mM glucose, the β cell activity was predominantly characterized by sustained oscillatory Ca^2+^ activity with oscillations being superimposed on an elevated basal Ca^2+^ level^27^. Typical filtered traces (29 Hz) from 5 cells within individual islet are shown in Fig. 5a–b for all four experimental groups. To quantify the intracellular Ca^2+^ activity in each subgroup we calculated the mean frequency and mean duration of individual Ca^2+^-oscillations. Overall, our results indicated a very high degree of inter-islet variability within subgroups in both mean frequency and duration of intercellular oscillations and no significant differences between any pair of groups could be detected, except for increased mean pulse duration in MT females (Supplementary Fig. 4). However, time courses of Ca^2+^ dynamics indicated that in both males and females, the traces were less synchronous in MTs compared to WTs (Fig. 5a,b). This observation was further corroborated by Supplementary Video 1 and 2, where the spatiotemporal behavior of Ca^2+^ activity is presented in a WT and a MT islet, respectively. To further quantify these visual assessments, we calculated the mean coactivity of all samples in each subgroup. Results shown in Fig. 5c revealed that the β cells were indeed significantly more synchronous in WT compared to MT islets.

In summary, in SNAP-25b-deficient islets there was decreased synchronization between β cells.

### SNAP-25b-deficiency results in segregated β cell networks

**Figure 6:**
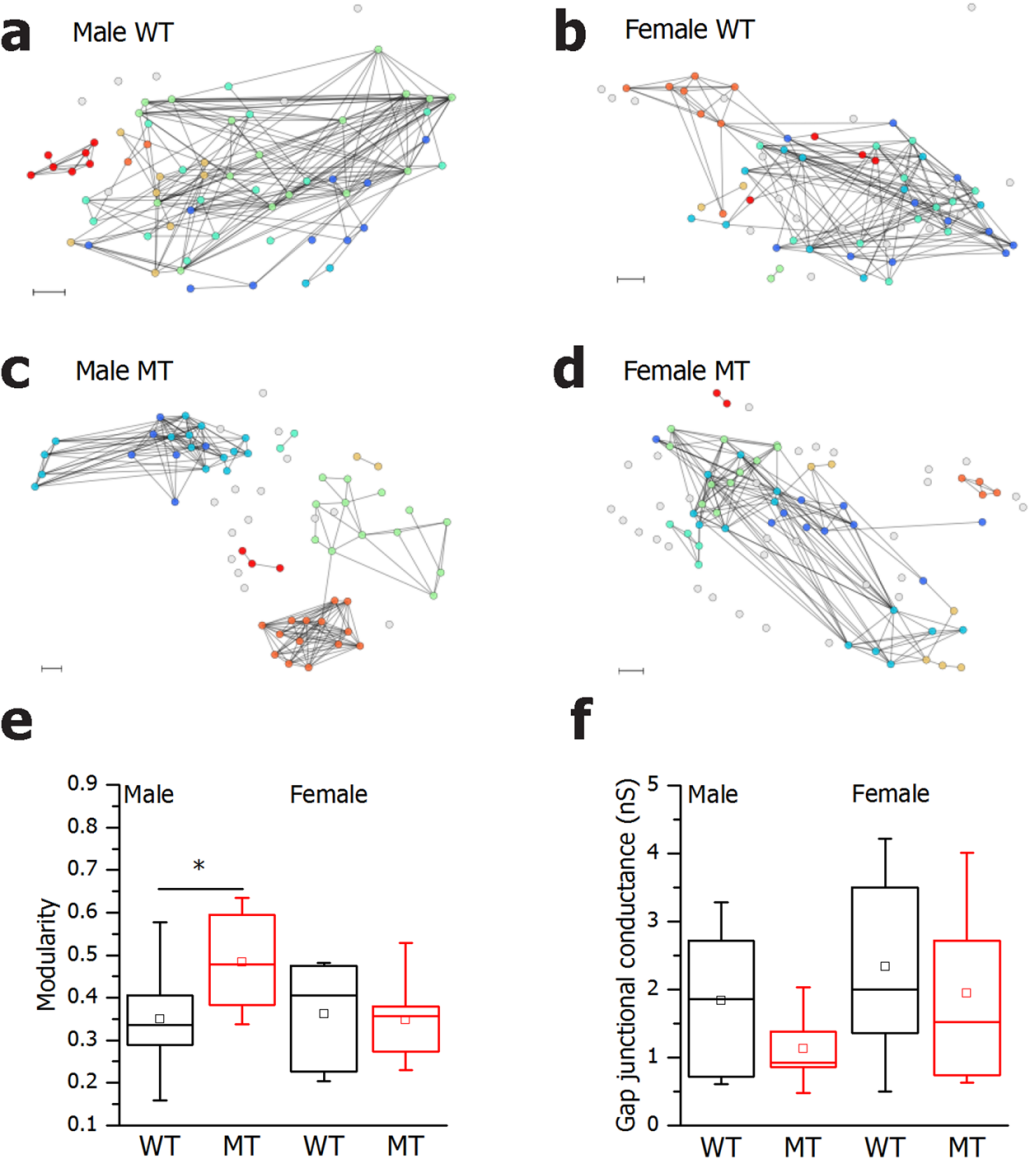
SNAP-25b-deficient β cells display disrupted β cell networks upon glucose stimulation. Representative functional β cell networks extracted on the basis of the synchronization level between a given pair of cells (**a–d**). Modularity of all functional networks in 12 week old males and females (**e**) WT males, *n* = 4, 8 islets, MT males, *n* = 4, 9 islets, WT females, *n* = 3, 11 islets, MT females, *n* = 5, 11 islets. Gap junction conductance in β cells during patch-clamp experiments (**f**) WT males, *n* = 2, 10 cells, MT males, *n* = 2, 11 cells, WT females, *n* = 4, 17 cells, MT females, *n* = 2, 11 cells. The box charts are defined in the same way as in Fig. 3. WT mice, black bars; MT mice red bars. Scale bar 10 µm. *P < 0.05.

The less regulated and faster Ca^2+^ responses together with impaired synchronization between β cells suggested lesions in the β cell network activity. The abovementioned observed morphological changes in islets of SNAP-25b-deficient mice implied possible alterations in the quality of cell-to-cell interactions which could contribute to the modified pattern of insulin release. β cells are electrically coupled and respond to glucose stimulation with a characteristic pattern of Ca^2+^-oscillations^27^. To assess the nature of intercellular communication between β cells, we analyzed their functional connectivity profiles. Four different functional networks from each subgroup are shown in Fig. 6a–d. In all extracted networks a modest degree of partition into sub-compartments was observed^36^. By calculating the modularity, we found that functional networks extracted from MT males were more segregated compared to WTs (Fig. 6e), while in females this phenomenon was not so pronounced. A more modular connectivity in the male MTs can be a consequence of β cell hyperplasia (Fig. 3a–b), accompanied by looser connections between β cells within such an enlarged islet. The gap junction conductance of β cells, measured by patch-clamp, tended to be decreased in MT males with a frequency distribution favoring weak connections and only few highly connected cells (Fig. 6f).

**Figure 7:**
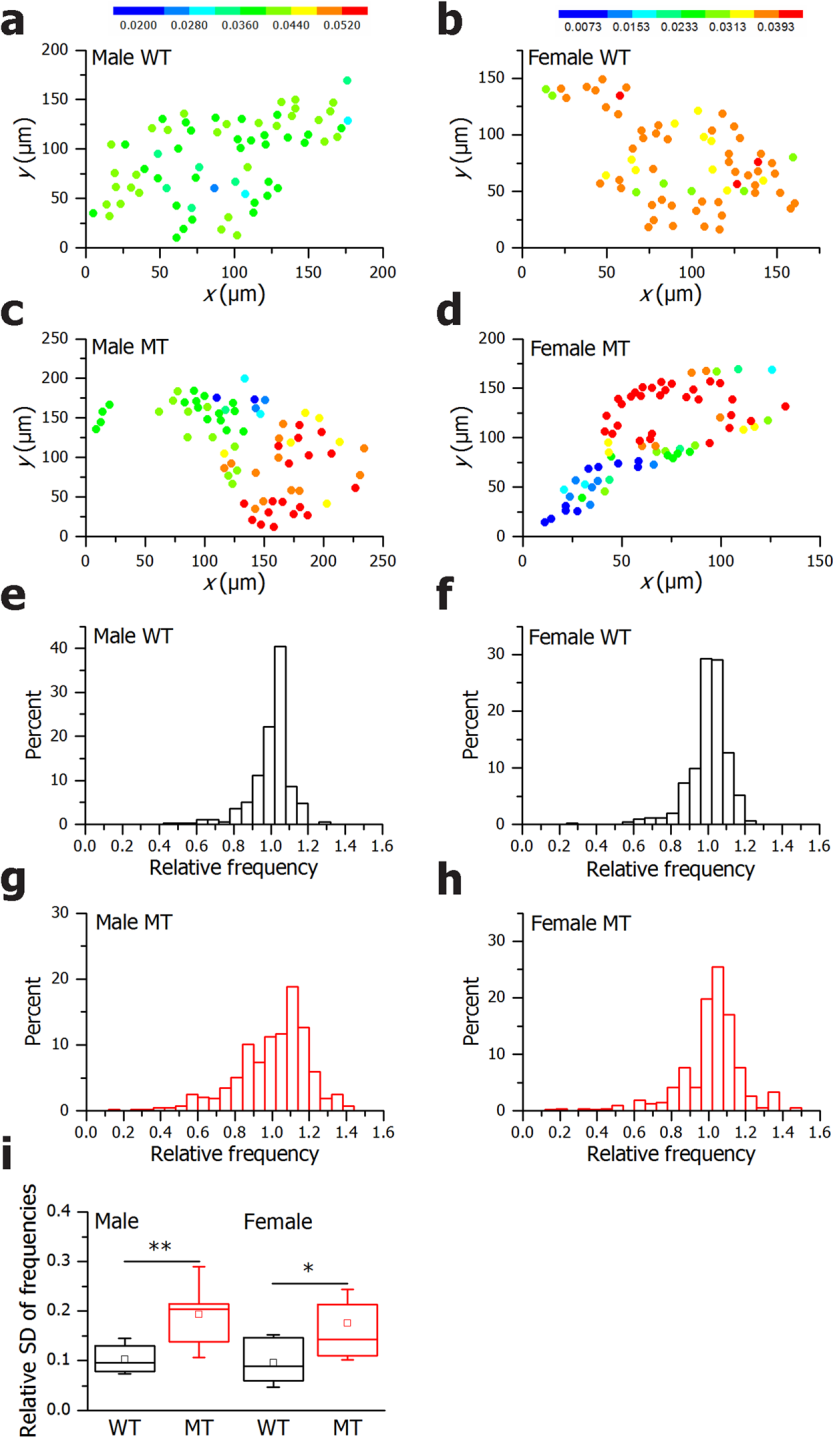
In SNAP-25b-deficient islets the spatiotemporal Ca^2+^ activity is less coordinated. The coordinates of β cells with color coded frequencies of individual cells in a typical 12 week old WT and MT male (**a** and **c**), WT and MT female (**b** and **d**). The color-bars express the frequencies in Hz for males and females, independently as shown in left and right panel. The distribution of relative frequencies are shown (scaled to the mean frequency in a given slice) for all cells in a given subgroup (**e**–**h**). In (**i**) the relative standard deviations of frequencies in individual slices for different subgroups in males and females are displayed. WT males, *n* = 4, 8 islets, MT males, *n* = 4, 9 islets, WT females, *n* = 3, 11 islets, MT females, *n* = 5, 11 islets. The box charts are defined in the same way as in Fig. 3. WT mice, black bars; MT mice red bars. *P < 0.05.

We additionally explored the spatial distribution and cell-to-cell variability of frequencies of Ca^2+^-oscillations. Figure 7a–d shows the position of cells with colors reflecting their frequencies. It can be observed that in both males and females, the frequencies were much more unified and homogeneously distributed in WTs compared to MTs. The dispersion of frequencies around the relative mean value is evidently broader in MTs than in WTs, thereby indicating that the spatiotemporal Ca^2+^ activity in MTs was less coordinated (Fig. 7e–h). To characterize this observation with a single quantity, we calculated the relative standard deviations of frequencies in all islets in each of the four subgroups. The results presented in Fig. 7i confirmed a significantly higher level of frequency heterogeneity in MT islets compared to WTs.

Taken together, SNAP-25b-deficient islets demonstrated impaired cell-to-cell communication between β cells.

## Discussion

This study shows that SNAP-25b-deficiency results in increased insulin release, higher number of β cells in males, altered islet size, lost collective control of Ca^2+^-oscillations and defective inter-β-cell-connectivity within islets, all features of the early phase of developing type 2 diabetes. Unexpectedly, the phenotype appears to mainly depend on less strict control of Ca^2+^ dynamics, resulting in premature influx of Ca^2+^ after stimulation and delayed lowering of [Ca^2+^]_*i*_ back to basal levels after glucose has been removed. The loss of accurate control of [Ca^2+^]_*i*_ likely leads to less synchronized Ca^2+^-oscillations and defects in cell-to-cell communication and in the long-term, prolonged increase of [Ca^2+^]_*i*_ can lead to apoptosis. Increased insulin secretion, concomitant with the loss of pulsatile release, is associated with development of insulin resistance and obesity^4^.

Dysregulation of insulin release from islets of Langerhans provides wide-ranging metabolic consequences for the entire body^1, 5^. We hypothesized that SNAP-25b-deficiency in islets primarily affected the core exocytotic SNARE machinery and thus the mechanism of insulin secretion. In the long-term, this could cause secondary effects, influencing β cell physiology, islet morphology, and differently affect insulin responsive tissues, thereby acting as a primary cause of the progressing metabolic disease^35^. SNAP-25b is the least abundant SNAP-25 isoform in the endocrine β cell^10, 11^, but insulin secretion from isolated SNAP-25b-deficient pancreatic islets was markedly increased, both after glucose stimulation and when challenged with KCl. As SNAP-25b-deficiency might facilitate vesicle fusion with the plasma membrane according to previous *in vitro* studies showing that less stable SNARE complexes are formed with SNAP-25a than SNAP-25b^12, 13^, we first explored this possibility in pancreatic slices using slow photo-release of caged Ca^2+^ in β cells. Surprisingly, we found no difference either in Ca^2+^-sensitivity, amplitude or rate of exocytosis in β cells between different experimental groups. If the increased insulin secretion from SNAP-25b-deficient β cells was not dependent on less stable exocytotic SNARE core complexes, the different SNAP-25 isoforms might act differently on upstream targets affecting insulin secretion. It is known that SNAP-25 interacts with VDCC and K^+^ channels, plasma membrane proteins regulating electrical activity and membrane potential^16, 18–20, 22^. Interaction between SNAP-25 and Kv2.1 in pancreatic β cells has previously been shown to increase insulin release^22^ and recently it was demonstrated that the SNAP-25b isoform together with syntaxin-1 was more efficient than SNAP-25a in inhibiting VDCC currents in chromaffin cells^20^. A possibility is that any effect on ion channels regulating Ca^2+^-influx also could affect the plasma membrane – endoplasmic reticulum interaction which is dependent on Ca^2+^ and phosphoinositide signaling^24^. Furthermore, in neuronal systems direct binding of SNAP-25 to Gβγ has been reported^37^ and G-protein-coupled-receptor activation can suppress conductance of VDCCs, and increases that of K^+^ channels in presynaptic terminals^38^. Similarly, G-protein-coupled-receptors can regulate the secretion of hormones from the islets of Langerhans^39^. Therefore, we investigated if SNAP-25b-deficiency affected intracellular Ca^2+^ dynamics in β cells. To assess the functionality of β cells by Ca^2+^-imaging we used pancreatic slices in which islets are surrounded by their natural environment, *i.e.* the exocrine pancreas. We found that a small population of β cells in MT islets responded to glucose prematurely and after lowering the glucose concentration, the MT male β cells showed a delayed deactivation time. This heterogeneity suggested that the strict cellular control of Ca^2+^ entry into the cytosol, either from extracellular sources or from intracellular stores was defective in MTs, which also was supported by the evidence that [Ca^2+^]_*i*_ oscillations were less synchronized between β cells. However, we cannot exclude that SNAP-25b-deficiency can have an effect on basal [Ca^2+^]_*i*_ as this technique allows only qualitative measurements. The impaired synchronization activity could by itself partly be dependent on lower gap junctional conductance in MT β cells as well as on the disrupted intra-islet β cell organization due to hyperplasia. We did notice a tendency of decreased gap junction conductance of β cells in SNAP-25b-deficient males, with a frequency distribution favoring weak connections and only few highly connected cells. In order to investigate if SNAP-25b-deficiency confers a direct or indirect effect on gap junction functionality on β cells in acute pancreatic slices, further experiments including suppression of both connexin36 and the different SNAP-25 isoforms have to be performed. However, these experiments are currently not technically possible in our experimental system. Recent evidence highlights the importance of inter-β-cell-connectivity within one islet, not only because its critical role in insulin release through the generation of coordinated rhythmic activity, but also because its sensitivity to insults. This connectivity can be disrupted by both environmental and genetic factors during the pathogenesis of type 2 diabetes^29–32, 34^. In previously described mouse models of prediabetes, less synchronized Ca^2+^-oscillations also have been related to increased insulin secretion elicited by glucose^26, 32, 33^. In our SNAP-25b-deficient mice we know that total pancreatic insulin content is increased, which might be part of a compensatory mechanism^35^. The increase in insulin secretion during prediabetes might be an attempt to overcome the loss of pulsatile release which is regarded as important for function of receptors located on target tissue^4^.

To further characterize the functional interactions between β cells upon glucose stimulation we used analytical tools from the theory of the complex network^40^. Previously, insights into the functional mechanisms and organization of pancreatic islets have been pointing out that β cell networks form heterogeneous, efficient, and clustered architectures^28, 36, 41, 42^. In the present study we could observe that β cells in SNAP-25b-deficient islets function in a more segregated manner upon glucose stimulation compared to the WT islets, explaining the less synchronized Ca^2+^ oscillations. These observations can be associated with large-scale disorganization of insulin release. This work also provides information on how males and females differently try to compensate for prediabetes. Female MTs were hyperglycemic and hyperinsulinemic, whereas male MTs developed β cell hyperplasia and larger islets. The dominance of preferably weak connections and segregated modules of β cells, accompanying β cell hyperplasia, can result in increased long-term insulin release. Regardless, similar dynamics in glucose-stimulated insulin secretion were observed in both sexes. It seems that MT females were more impaired compared to males, maybe closer to turning the prediabetes phenotype into a full-blown type 2 diabetes^5, 35^. It is noteworthy that islets from WT females had an increased number of β cells compared to WT males, but that was not associated with increased insulin secretion or changed islet area, as females had smaller β cells. WT females showed increased insulin sensitivity compared to males probably due to different body composition. These responses might be attributed to the compensation of energy demands females would encounter in case of pregnancy^43^.

Type 2 diabetes is a multifactorial, often polygenetic disease but most of the identified risk genes affect β cell physiology and function^44^. In addition, several polymorphisms have also been identified in genes that affect insulin secretion or metabolic parameters, such as the L-type calcium channel, the K_ATP_ channel, G-protein coupled receptors and syntaxin^33, 45–48^. Furthermore, SNPs in the human gene for *Snap25* have been associated with glycemic parameters or severity of metabolic syndrome in type 2 diabetes patients^49, 50^. In this context it is noteworthy, that the metabolic phenotype in our SNAP-25b-deficient mice became severely worsened in combination with a Western diet intervention^35^. Given that SNAP-25a and SNAP-25b isoforms confer different strength to complex molecular interactions there is a possibility that SNAP-25b-deficiency, or changed expression ratio of the two spliced variants, can in a long-term perspective contribute to development of disease in humans.

In conclusion, our results suggest that SNAP-25b plays an essential role in keeping a tight control of insulin secretion, not only by mediating the release process, but also via regulating Ca^2+^ dynamics and collective control of Ca^2+^-oscillations. A detailed investigation of how SNAP-25a and SNAP-25b, alone or in combination with syntaxin, differently interact with separate ion channel subunits, G-protein coupled receptors or other proteins in β cells, is required to fully understand the mechanism behind the impaired Ca^2+^ handling in SNAP-25b-deficient mice. Our findings that the regulation of Ca^2+^ dynamics and insulin secretion are controlled via different isoforms of SNAP-25, at the level of electrical activity, further underlines the importance of considering SNARE protein function outside the context of late exocytotic events. Hopefully this will provide new understandings of how type 2 diabetes can develop and enable the identification of new therapeutic targets.

## Methods

### Animals

Generation of SNAP-25b-deficient mice back-crossed on C57BL/6Crl, breeding, maintenance and genotyping were performed as previously described^12^. SNAP-25b-deficient animals (MT) and wild-type (WT) littermates were euthanized either by CO_2_ or cervical dislocation. Animal studies were done in accordance with the guidelines from the local authorities, *i.e*., the Stockholm Northern Animal Experiments Ethics Board, following the approval of the Administration of the Republic of Slovenia for Food Safety, Veterinary Sector and Plant Protection (Permit number: 34401–61–2009/2, 34401–46/2014/4, 34401–12/2015/3) and in accordance with Directive 2010/63/EU of the European parliament and of the Council on the Protection of Animals Used for Scientific Purposes.

### Islets isolation

After sacrifice, abdomen was accessed via laparotomy and collagenase P (1 mg/ml) (Roche, Mannheim, Germany) diluted in HBSS pH 7.4 (Thermo Scientific, Waltham, MA) was injected into the proximal common bile duct clamped distally at the major duodenal papilla of Vater. After injection, the pancreas was extracted and incubated in HBSS at 37 °C for 30 min without shaking. Ice-cold HBSS (without BSA) was added and 1–2 strokes with 18 G needle was applied to dislodge islets attached to the tissue. After 4 washes (2 with HBSS without BSA and 2 with HBSS 0.5% BSA) islets were hand-picked under a stereo microscope (KL200 LED, Leica, Wetzlar, Germany). Purified islets were transferred into petri dishes containing RPMI-1640 (Thermo Scientific, Waltham, MA, USA) with a final concentration of heat inactivated fetal bovine serum (10%), glutamine (2 mM), penicillin (100 U/ml) and streptomycin (100 μg/ml) (Thermo Scientific, Waltham, MA, USA) and incubated at CO_2_ (5%) and 37 °C overnight.

### Insulin secretion assay and insulin measurement

After overnight incubation, approximately 80 islets from each pancreas were transferred to a chromatograph column (PERI-4.2, BioRep technologies, Miami Lakes, FL, USA) filled with Bio-Gel P-4 (Bio-Rad Laboratories, Hercules, CA, USA) to stabilize them during the perifusion. Islets were pre-perifused with NaCl (125 mM), KCl (5.9 mM), CaCl_2_ (1.28 mM), MgCl_2_ (1.2 mM), HEPES (25 mM), BSA (0.1%) and glucose (3 mM), pH 7.4 for 45 min at 37 °C. The islets were perifused in the buffer above for 12 min, then sequentially exposed to 11 mM glucose for 35 min followed by 3 mM glucose for 15 min and the protocol finished with 25 mM KCl + 3 mM glucose for 15 min to empty all possible insulin granules. Fractions (50 μl) of the perifusate were collected every min during stimulation in a 96-well plate. The collected fractions were then measured for insulin concentration by the AlphaLISA detection kit (PerkinElmer, Waltham, MA, USA) with a plate reader (EnVision2103, PerkinElmer, Waltham, MA, USA).

### Glucose tolerance test and serum insulin levels

Mice were starved overnight for 12 h. One hour before glucose injection, EMLA cream (25 mg lidocaine and 25 mg prilocaine, AstraZeneca, London, United Kingdom) was applied on the tail (to minimize stress) and after 15 min blood glucose was measured. After 45 min mice were injected intraperitoneally with glucose (2 g/kg body weight) and blood glucose levels were determined at 0, 2.5, 5, 7.5, 10, 15 min using a FreeStyle Glucometer (Abbott Diabetes Care, Witney, United Kingdom). Blood was centrifuged for 20 min, 10,000 × g at 4 °C and serum was collected and frozen at −80 °C until use. Serum insulin levels were analyzed using an ultrasensitive mouse insulin ELISA kit (Crystal Chem Inc., Downers Grove, IL, USA). The AUC was calculated using the basal levels of blood glucose and serum insulin as baselines (baselines used for calculations are indicated with dotted lines) and the HOMA_IR_ was calculated using the formula: fasting insulin (mU/L) × fasting blood glucose (mmol/L)/22.5.

### Immunohistochemistry

Mice were transcardially perfused and the pancreas was processed as described previously^12^. Mounted 16 μm-thick sections were incubated with primary antibodies in a humidified chamber at 4 °C overnight (rabbit anti-glucagon antibody, 1:1,000, BioGenex, Fremont, CA, USA) and guinea pig anti-insulin antibody (1:200, Bio-Yeda, Rehovot, Israel). After washing in PBS (0.01 M) they were incubated with secondary antibodies for 90 min at room temperature (donkey Cy3-conjugated anti-rabbit IgG and donkey FITC-conjugated anti-guinea pig IgG, 1:150 and 1:40 respectively, Jackson Immunoresearch Europe, Suffolk, United Kingdom). Sections were finally incubated with DAPI (1:10,000, Bio-Rad, Hercules, CA, USA) diluted in PBS for 15 min at room temperature and mounted using 2.5% DABCO in glycerol (Sigma-Aldrich, ST. Louis, MO, USA) and stored at −20 °C in darkness. Another set of sections was used for H&E staining (Histolab, Gothenburg, Sweden) and mounted with VectaMount permanent mounting medium (Vector Laboratories Inc., Burlingame, CA, USA). For the detection of apoptotic β cells, sections were processed as according to the commercial kit used (Click-iT Plus TUNEL assay, Thermo Scientific, Waltham, MA) and co-labeled with insulin.

### Image acquisition and analysis

The pancreatic sections were examined with Nikon Eclipse E600 fluorescence microscope with objective lenses 20 × (Nikon, Tokyo, Japan) equipped with appropriate filters and ORCA-ER, C4742–80 digital camera (Hamamatsu Photonics K.K., Shizuoka, Japan), using Hamamatsu Photonics Wasabi 150 software. Images were also acquired by use of upright laser scanning confocal microscope based on a Leica TCS-SP5 II (Leica Microsystems, Wetzlar, Germany), together with long-distance water-dipping objectives (Leica HXC-APO 20 × /0.5, Wetzlar, Germany), and a Leica LAS software (Leica, Wetzlar, Germany). For insulin- and glucagon-expressing cell quantifications, one randomly-chosen pancreatic section/animal was used (*n* = 4 animals in each experimental group, in each section three islets between 0.1 and 0.02 mm^2^ were analyzed), each insulin/glucagon positive cell with detectable nucleus was counted and the number of cells was divided by the islet area. Two randomly-chosen H&E stained pancreatic sections/animal were used (*n* = 4 for all experimental groups) for islet size measurement and number of islet per section. Around 400 islets were counted. All measurements were done with the ImageJ program (National Institutes of Health, Bethesda, MD, USA). For the detection of apoptotic β cells, one randomly-chosen pancreatic section/animal was used (*n* = 3 animals in each experimental group) and all islets within a section were analyzed.

### Tissue slice preparation

Tissue slices were cut from pancreata of 12 week old WT and MT mice as previously described^51^. Briefly, animals were sacrificed by cervical dislocation and the abdomen was accessed via laparotomy. A low-melting point agarose (1.9%, Lonza Rockland Inc., Rockland, ME, USA) in extracellular solution, ECS, consisting of: NaCl (125 mM), NaHCO_3_ (26 mM), glucose (6 mM), lactic acid (6 mM), myo-inositol (3 mM), KCl (2.5 mM), Na-pyruvate (2 mM), CaCl_2_ (2 mM), NaH_2_PO_4_ (1.25 mM), MgCl_2_ (1 mM), ascorbic acid (0.5 mM) at 40 °C was injected into the proximal common bile duct clamped distally at the major duodenal papilla of Vater. Immediately thereafter, the pancreas was cooled using ice-cold ECS and the agarose-permated pancreas was extracted and gently washed with ice-cold ECS. Small tissue blocks (0.1–0.2 cm^3^ in size) were cut and plunged into the agarose at 40 °C. Individual cubes of cooled agarose containing tissue blocks were glued (Super Attak, Henkel Slovenija d.o.o., Maribor, Slovenia) onto the sample plate of the VT 1000 S vibratome (Leica, Nussloch, Germany). The tissue was cut at 0.05 mm s^−1^ at 70 Hz into 140 μm-thick slices of a surface area of 20–100 mm^2^. Throughout preparation and during slicing the tissue was held in an ice-cold ECS continuously bubbled with a gas mixture containing O_2_ (95%) and CO_2_ (5%) at barometric pressure to ensure oxygenation and a pH of 7.4. After cutting the slices were collected in 30 ml of HEPES-buffered saline, HBS, consisting of: NaCl (150 mM), HEPES (10 mM), glucose (6 mM), KCl (5 mM), CaCl_2_ (2 mM), MgCl_2_ (1 mM); titrated to pH 7.4 at room temperature. All chemicals were obtained from Sigma-Aldrich (St. Louis, MO, USA) unless indicated.

### Electrophysiology measurements

Patch pipettes were pulled from borosilicate glass capillaries (GC150F-15, Harvard Apparatus, Holliston, MA, USA) using a horizontal pipette puller (P-97, Sutter Instruments, Novato, CA, USA). The pipette resistance was 2–3 MΩ in Cs^+^-based solution. Fast pipette capacitance (Cfast) was compensated in cell-attached mode, slow membrane capacitance (Cslow) and series conductance (Gs) were compensated after establishment of whole-cell mode. Only experiments with Gs > 50 nS were proceeded. Recordings were performed in the standard whole-cell mode via a patch-clamp lock-in amplifier (SWAM IIc, Celica, Slovenia) connected to a PC via A/D converter (16 bit, NI USB-6343, X Series Multifunction DAQ, National Instruments, Austin, TX, USA) and recorded on the PC hard disk using WinWCP V5.1.6 software (John Dempster, University of Strathclyde, United Kingdom). The same software was used for identifying β cells by their Na^+^ current inactivation properties and for measuring membrane capacitance change after slow photo-release of caged Ca^2+,^ ^52^. Latter was performed as described previously^53^. A continuous sine voltage (1600 Hz, 11 mV RMS amplitude) was applied to measure Cm, a parameter that is proportional to membrane surface area^54^. Resting membrane potential in voltage-clamp mode was −80 mV. The pipette solution used for Ca^2+^-induced capacitance and current measurements was composed of NP-EGTA (5 mM), CaCl_2_ (4 mM), mM Fura 6 F (0.1) (Invitrogen, Eugene, OR) together with CsCl (125 mM), HEPES (40 mM), MgCl_2_ (2 mM), TEA–Cl (20 mM), Na_2_ATP (2 mM) at pH 7.2 and osmolality 300 ± 10 mOsm. Signal processing and curve fitting was done using Matview (Wise Technologies, Ljubljana, Slovenia) and MATLAB (The MathWorks, Inc., Natick, MA, USA).

### Loading of dyes

For [Ca^2+^]_*i*_ imaging 8–10 slices were incubated in a petri dish (5 ml) filled with of HBS (3.333 ml) containing Oregon Green 488 BAPTA-1 acetoxymethyl ester calcium fluorescent dye (6 μM, OGB-1, Invitrogen, Eugene, OR, USA), Pluronic F-127 (0.03% w/v) and dimethylsulphoxide (DMSO, 0.12% v/v) for 50 minutes on an orbital shaker (50 turns min^−1^) at room temperature and protected from light^55^.

### Measurements of cytoplasmic free Ca^2+^ activity

Ca^2+^ imaging was performed on a Leica TCS SP5 AOBS Tandem II upright confocal system using a Leica HCX APO L 20x water immersion objective (NA = 1.0). OGB-1 was excited by an argon 488 nm laser and the emitted light was detected by Leica HyD hybrid detector in the range of 500–700 nm (all from Leica Microsystems GmbH, Wetzlar, Germany). The slices were fixed to the bottom of the bath chamber by U-shaped platinum frame with nylonfibers and were continuously perifused with the bubbled ECS at 35–37 °C containing 6 and 12 mM glucose, respectively. To avoid recording from cells in the damaged cut surface, cells lying at least 15 μm below the surface were imaged. To determine the delays in the onsets in the [Ca^2+^]_*i*_ after beginning of the stimulation with 12 mM glucose and the delays in deactivation in the [Ca^2+^]_*i*_ response after the end of the stimulation with 12 mM glucose images were acquired at a spatial resolution of 512 × 512 pixels and a temporal resolution of 1 Hz. The total time for 12 mM glucose stimulation was 15–22 min. To determine the frequencies and the durations of calcium oscillations and for the network analysis, images were acquired for 5 min at a spatial resolution of 256 × 256 pixels and a temporal resolution of 29 Hz. Regions of interest were selected based on higher spatial resolution (1024 × 1024) reference images. Time traces were analyzed off-line from regions of interest employing Leica Application Suite Advanced Fluorescence software (Leica Microsystems GmbH, Wetzlar, Germany), exported and further analyzed to determine the delays in the onsets and delays in deactivation in the [Ca^2+^]_*i*_ response using MATLAB (The MathWorks, Inc., Natick, MA, USA). Traces were corrected for photobleaching of the dye employing a combination of linear and single exponential fit as described previously^27^ and signals expressed as (F – F0)/F0 ratios, where F0 is the initial fluorescence intensity and F is the fluorescence signal recorded at an individual time point during the experiment. All recorded time series were digitally band-pass filtered in order to remove noise and artefacts, and then additionally smoothed with an adjacency averaging procedure. To calculate the frequencies and durations of Ca^2+^ oscillations the onsets and the endings of individual oscillations were defined as the time in which the signal decreases bellow the half of the maximal amplitude in the given oscillation^27^. In this manner the activity profiles of all cells were binarized, whereby the time between the onset and ending of an oscillation was denoted as 1, whilst 0 otherwise. The cells with a low signal-to-noise ratio in which a firm binarization of the signal was not possible were excluded from further analyses.

### Synchronization and functional connectivity of β cells

The level of synchronization among β cells was determined on the basis of binarized Ca^2+^ activity by means of the coactivity matrix^56^, whose *ij*-th element is defined as follows: $Cij=\frac{Tij}{\sqrt{TiTj}}$

and reflects synchronization between the *i*-th and the *j*-th cell. In Eq. (1) $Tij$ stands for the total coactivity time in which both cells were simultaneously active and *Ti* and *Tj* are the total individual activity time for both cells. If *Cij=0* then no correlation between the *i*-th and *j*-th cells exists, whilst *Cij=1* signifies completely synchronous and aligned dynamics. To describe the global level of synchronization in the whole slice, the mean coactivity was calculated by averaging over all cell pairs. Supplementary Fig. 5 schematically presents how the recorded time series were processed and how the synchronization between β cells was evaluated.

To get a more detailed insight into the intercellular interaction patterns functional connectivity maps were constructed. Two cells were considered to be functionally connected if their activity profiles showed a high enough degree of synchronization, *i.e.Cij* exceeded a given threshold value. A similar methodology was used for extraction of the functional connectivity patterns elsewhere^36, 56, 57^. In this study a variable connectivity threshold was used in order to ensure that all examined β cell networks had the same mean number of connections per cell – 6. The segregation of β cell networks was evaluated by calculating a global metric called “modularity”, as proposed by Blondel *et al*.^58^. A higher modularity indicated a high degree of sub-compartmentalization, whereas close-to-zero values indicated an integrated and a module-free network^36, 59^.

### Statistical analysis

All statistical analyses were done using GraphPad Prism (GraphPad Software, San Diego, CA, USA). Two-way ANOVA followed by Bonferroni multiple comparisons test; Mann-Whitney and Student’s *t* test were used to verify statistically significant differences in all our experiments dependently on non-Gaussian or Gaussian distribution, respectively. The level of significance was set at a *P* value < 0.05.

## Electronic supplementary material

Supplementary Information

Supplementary Video 1

Supplementary Video 2
