# Supplementary material for: SNAP-25b-deficiency increases insulin secretion and changes spatiotemporal profile of Ca2+oscillations in β cell networks
Source: Sci Rep. 2017 Aug 10;7:7744. doi: 10.1038/s41598-017-08082-y (PMC5552776; doi:10.1038/s41598-017-08082-y)
Supplement: Supplementary file 1 — Supplementary Information [file 41598_2017_8082_MOESM1_ESM.pdf]

Supplementary information

SNAP-25b-deficiency increases insulin secretion and changes spatiotemporal profile of Ca^{2+}oscillations in β cell networks

Teresa Daraio[1], Lidija Križančić Bombek[2], Marko Gosak[2,3], Ismael Valladolid-Acebes[1], Maša Skelin Klemen[2], Essam Refai[1], Per-Olof Berggren[1], Kerstin Brismar[1], Marjan Slak Rupnik[2,4,*], Christina Bark[1,*].

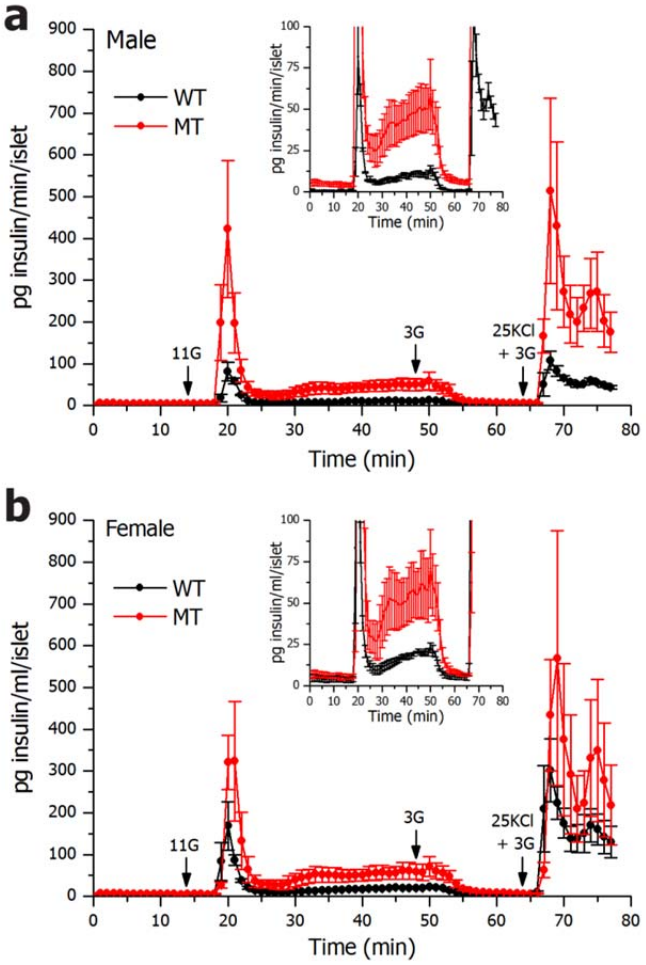

Supplementary Figure 1: Islets isolated from SNAP-25b-deficient mice secrete more insulin in response to glucose. Glucose-induced insulin release was measured in isolated pancreatic islets from 12 week old male (A) and female (B) mice. Insets represent magnifications of the second phase of insulin secretion. *n*=3-4 animals per group. Data are represented as mean ± SEM. WT mice, black lines; MT mice red lines.

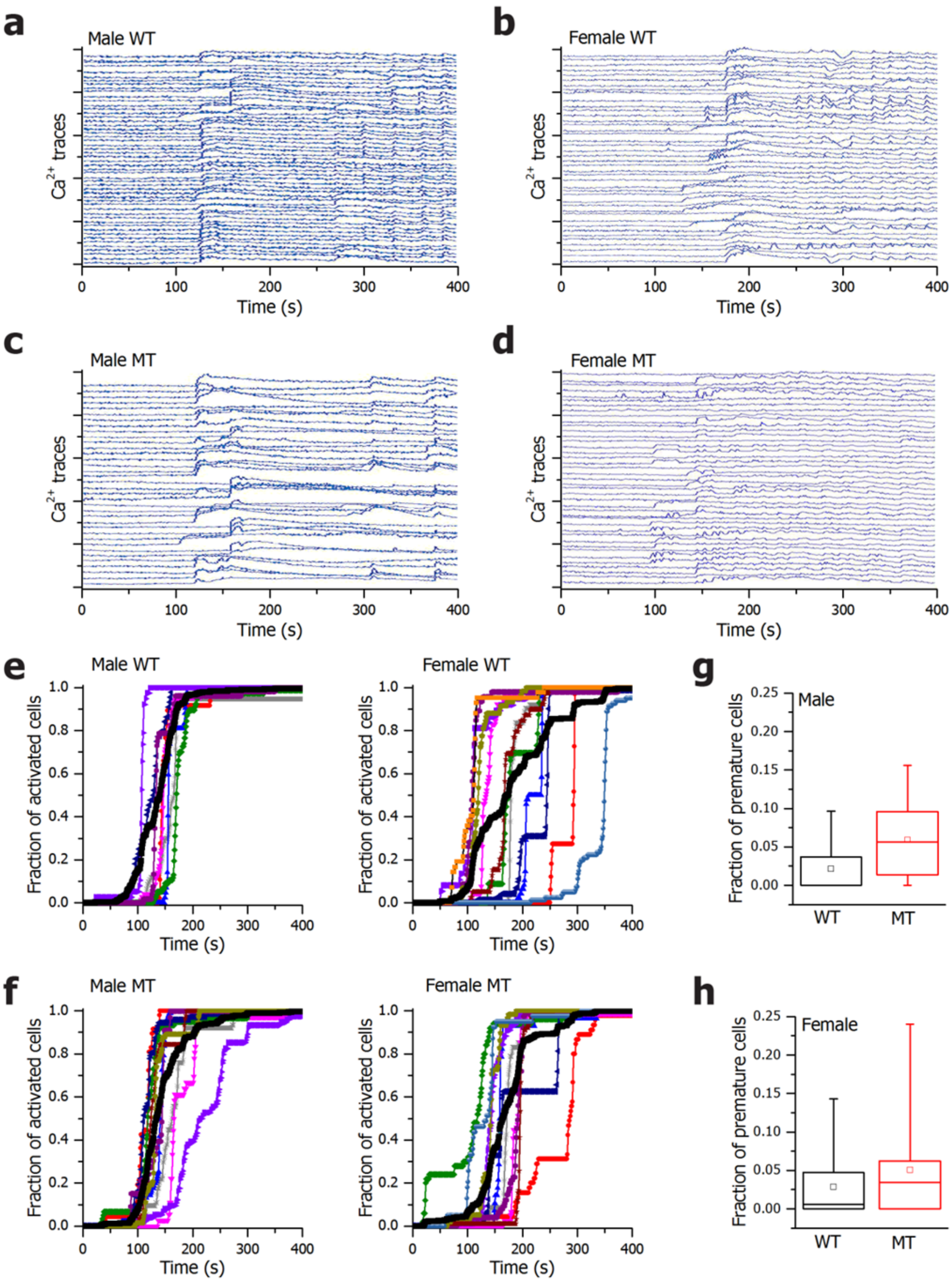

Supplementary Figure 2: Glucose-stimulated activation of β cells. Recorded $[Ca^{2+}]_i$ traces in the phase of activation of β cells in a typical 12 week old WT and MT male (A and C) and WT and MT female (B and D). At time 0 the glucose concentration changed from 6 to 12 mM. Cumulative activation of β cells in individual slices after the rise in glucose concentration in given sub-groups (E and F). Colored lines with small symbols denote individual slices and the thick black line signifies the mean response in a given sub-group (E and F). Fraction of premature cells activated before the 70% of the mean activation value within a given islet (G and H). WT males, *n*=4, 8 islets, MT males, *n*=4, 10 islets, WT females, *n*=3, 12 islets, MT females, *n*=5, 11 islets. The box charts are defined in the same way as in Figure 3. WT mice, black bars; MT mice red bars.

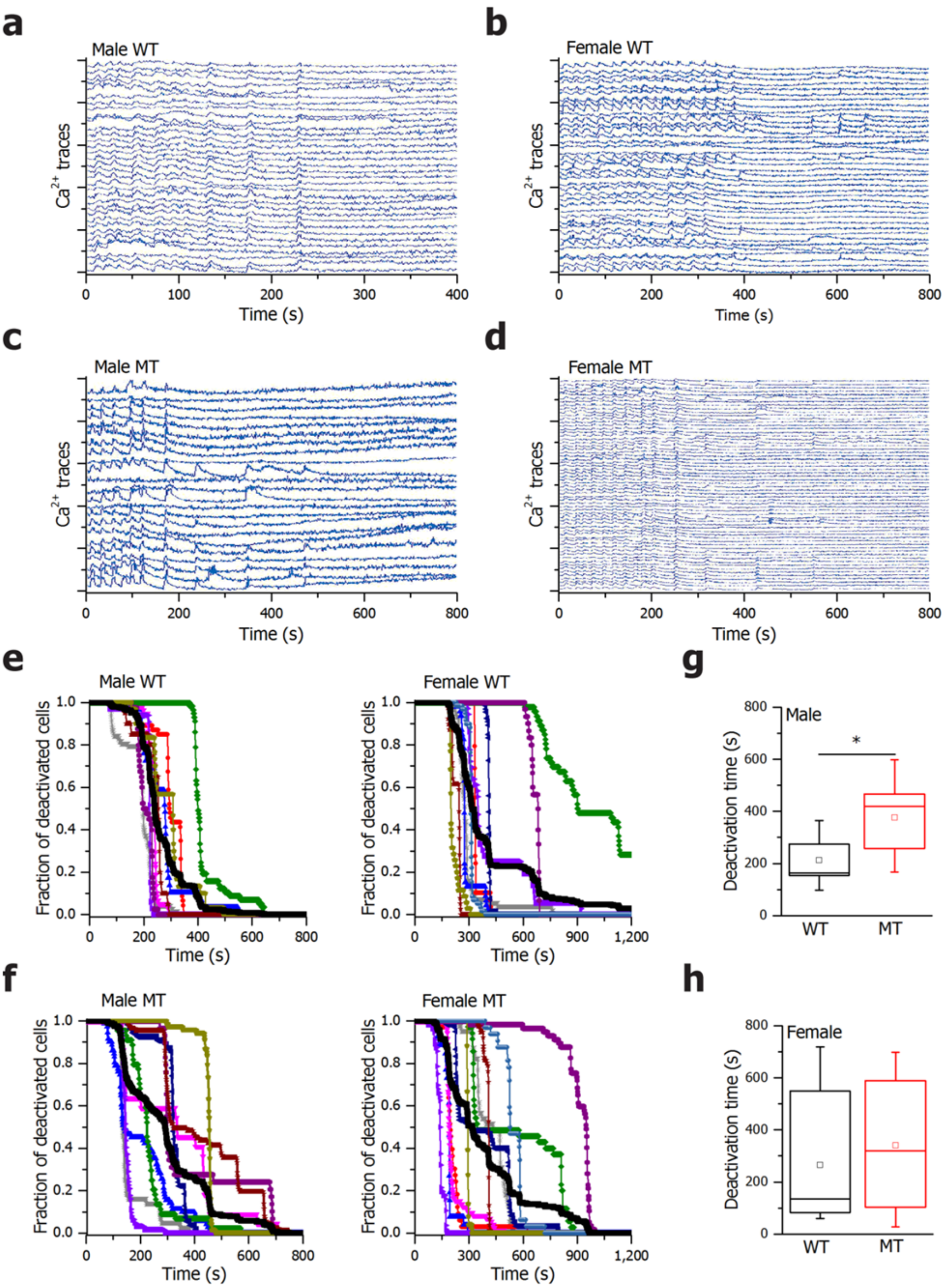

Supplementary Figure 3: Deactivation of β cells. Recorded $[Ca^{2+}]_i$ traces in a typical 12 week old WT and MT male (A and C) and WT and MT female (B and D) after lowering the glucose concentration. At time 0 the glucose concentration changed from 12 mM to 6 mM. Cumulative deactivation in β cells, in individual slices, from the end of stimulation in all given sub-groups (E and F). Colored lines with small symbols denote individual slices and the thick black line signifies the mean response in a given sub-group. Comparison of deactivation times between WTs and MTs, defined as the time between the first and the last cell become inactive (G and H). WT males, *n*=4, 8 islets, MT males, *n*=4, 9 islets, WT females, *n*=3, 11 islets, MT females, *n*=5, 11 islets. The box charts are defined in the same way as in Figure 3. WT mice, black bars; MT mice red bars. *$P<0.05$.

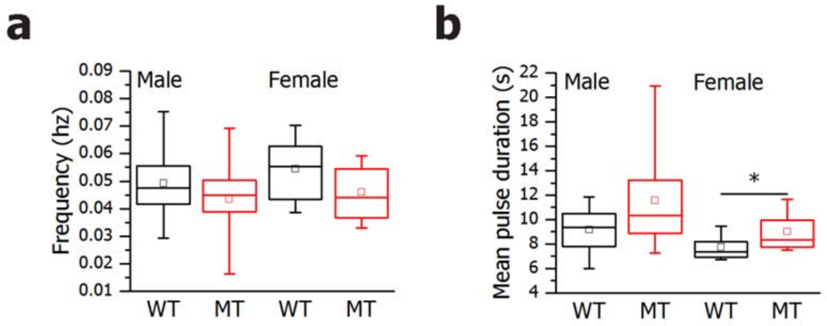

Supplementary Figure 4: Intracellular characteristics of Ca^{2+} signaling in β cells. The islet mean frequency of Ca^{2+}-oscillations in 12 week old males and females (A) and the islet mean durations of individual Ca^{2+}-oscillations for males and females (B). The intracellular oscillations exhibit a high level of inter-islet-variability with periods of oscillations ranging from 1 min^{-1} to 4.7 min^{-1} and individual oscillation durations spanning from 6 to 21 sec. WT males, *n*=4, 8 islets, MT males, *n*=4, 9 islets, WT females, *n*=3, 11 islets, MT females, *n*=5, 11 islets. The box charts are defined in the same way as in Figure 3. WT mice, black bars; MT mice red bars. *$P<0.05$.

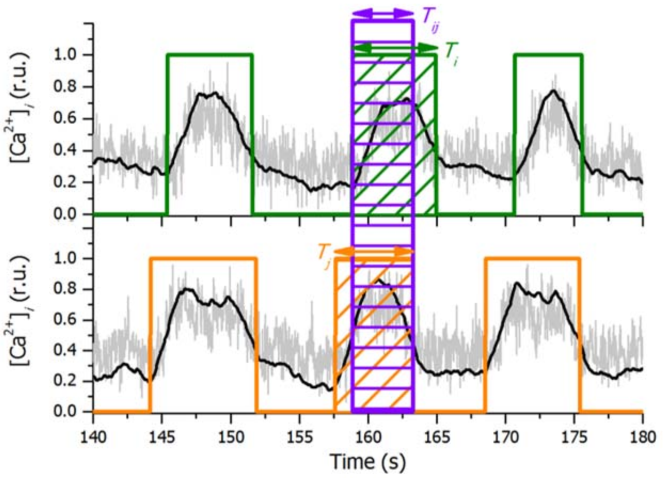

Supplementary Figure 5: Quantification of the measured Ca^{2+} signals. The grey lines represent the recorded Ca^{2+} traces of two active β cells (upper trace cell 1, lower trace cell 2). Recordings were band-pass filtered to remove noise, baseline trends and artefacts, and then smoothed with an adjacency averaging procedure. The obtained traces (black line) were then accordingly binarized. The cells with a low signal-to-noise ratio in which a firm binarization of the signal was not possible, were excluded from further analyses. The binarized activity profiles were used for the calculation of the frequencies and durations of Ca^{2+}-oscillations, and the level of synchronization among β cells by means of the coactivity coefficient, that measures the overlap of activity (T_{ij}, violet area) of *i-th* (T_i, green area) and *j-th* (T_j, orange area). Accordingly, the coactivity coefficient between the pair of Ca^{2+} traces is defined as

$$Cij = \frac{Tij}{\sqrt{TiTj}}$$

Supplementary Video 1: Animation of binarized spatiotemporal Ca^{2+}-activity during 12 mM glucose stimulation in a typical WT male sample.

Supplementary Video 2: Animation of binarized spatiotemporal Ca^{2+}-activity during 12 mM glucose stimulation in a typical MT male sample.
